# Endocrine Disrupting Chemicals Mediated through Binding Androgen Receptor Are Associated with Diabetes Mellitus

**DOI:** 10.3390/ijerph15010025

**Published:** 2017-12-23

**Authors:** Sugunadevi Sakkiah, Tony Wang, Wen Zou, Yuping Wang, Bohu Pan, Weida Tong, Huixiao Hong

**Affiliations:** 1 National Center for Toxicological Research, Food and Drug Administration, Jefferson, AR 72079, USA; Suguna.Sakkiah@fda.hhs.gov (S.S.); Wen.Zou@fda.hhs.gov (W.Z.); Yuping.Wang@fda.hhs.gov (Y.W.); Bohu.Pan@fda.hhs.gov (B.P.); Weida.Tong@fda.hhs.gov (W.T.); 2 Department of Biology, Arkansas University, Fayetteville, AR 72701, USA; tonywang@email.uark.edu

**Keywords:** androgen receptor, diabetes mellitus, metabolic syndrome, cancer, androgenic activity compounds

## Abstract

Endocrine disrupting chemicals (EDCs) can mimic natural hormone to interact with receptors in the endocrine system and thus disrupt the functions of the endocrine system, raising concerns on the public health. In addition to disruption of the endocrine system, some EDCs have been found associated with many diseases such as breast cancer, prostate cancer, infertility, asthma, stroke, Alzheimer’s disease, obesity, and diabetes mellitus. EDCs that binding androgen receptor have been reported associated with diabetes mellitus in in vitro, animal, and clinical studies. In this review, we summarize the structural basis and interactions between androgen receptor and EDCs as well as the associations of various types of diabetes mellitus with the EDCs mediated through androgen receptor binding. We also discuss the perspective research for further understanding the impact and mechanisms of EDCs on the risk of diabetes mellitus.

## 1. Diabetes Mellitus

Metabolic diseases have dramatically increased over the last several decades. Diabetes mellitus are described as a complex and serious condition of metabolic disease associated with abnormally high levels of blood sugar (glucose) resulting from deficiency of insulin secretion and/or action. Generally, there are two types of diabetes mellitus. Type 1 diabetes mellitus (T1DM) is an autoimmune condition in which the immune system is activated to destroy the cells in the pancreas which produce insulin. Type 2 diabetes mellitus (T2DM) is a progressive condition in which the body becomes resistant to the normal effects of insulin and/or gradually loses the capacity to produce enough insulin in the pancreas [1]. Both types are complex and serious. The annual incidence of both T1DM and T2DM in the United States has significantly increased [2]. Diabetes mellitus is recognized as the biggest epidemic disease for the public health of the 21st century. Currently, there are more than 340 million people worldwide affected by diabetes mellitus and this number is projected to be double by the year 2025 [3]. In 2013, 1.5 million diabetes mellitus patients ended their life in the world. In the U.S. alone, about 30.3 million people of all age, 9.4% of the U.S. population, had diabetes mellitus in 2015 and about 1.5 million new cases of diabetes mellitus were diagnosed among adults aged 18 years or older [4]. The total direct and indirect estimated cost of diagnosed diabetes mellitus in the U.S. in 2012 was $245 billion [5]. Therefore, it is imperative to understand the factors underling of this emerging metabolic disorder to prevent its deleterious impact on the public health and economic development.

The mechanisms of diabetes mellitus have not been fully understood. Several pathogenic processes are involved in the development of diabetes mellitus. Type 1 diabetes mellitus is resulting from autoimmune destruction of the β-cells of the pancreas with consequent insulin deficiency to abnormalities that result in resistance to insulin action [6]. Type 2 diabetes mellitus is a more complex hormone secretion and metabolic disorders. Overweight and obesity are considered the major contributions to insulin dysfunction through various biological pathways [7]. Roles of environmental chemicals in the development of diabetes mellitus have also been extensively investigated [8,9]. For instance, some endocrine disrupting chemicals (EDCs) such as bisphenol A, 2,3,7,8-tetrachlorodibenzo-*p*-dioxin, diethylhexyl phthalate and polychlorinated biphenyl have been proven to induce insulin resistance in cellular and animal models [10,11], leading to the “diabetogen hypothesis”. The diabetogen hypothesis proposes that every EDC circulating in plasma able to produce insulin resistance, independently of its obesogenic potential and its accumulation in adipocytes, may be considered a risk factor for metabolic syndrome and T2DM.

It has been reported that sex-specific activation of androgen receptor (AR) in the hypothalamus, skeletal muscle, liver, adipose tissue and pancreatic islet β cells accounts for maintenance or disruption in energy metabolism and glucose homeostasis [12]. Men with testosterone deficiency are at increased risk for T2DM [13]. Navarro et al. revealed that testosterone enhances glucose-stimulated insulin secretion via the interaction between an extranuclear AR and the glucagon-like peptide-1 (GLP-1) receptor in β cells, which increases cellular cyclic adenosine monophosphate (cAMP) levels and amplifies the incretin effect of GLP-1 [14].

## 2. Target Receptors of Endocrine Disrupting Chemicals

Nuclear receptors are the ligand-regulated transcriptional factors that play a key role in the development and homeostasis, reproductive and metabolic disorders like diabetes mellitus, obesity, and cancer [15]. Hence, nuclear receptors are considered as major drug targets in the drug discovery process. All nuclear receptors contain three major domains: N-terminal domain (NTD), DNA-binding domain (DBD), and ligand-binding domain (LBD). They have a similar 3D structure of the ligand-binding domain which consists of 12 helices. However, the hydrophobic pocket size (active site) is significantly different among nuclear receptors [16]. This hydrophobic pocket is formed by H3, H7 and H10. The H12 functions as a movable lid at the entrance of the ligand binding pocket [17]. Any small molecule that binds in the active site of the ligand binding domain undergoes a conformational change in the AF2 site. There are over 100 members in the nuclear receptors superfamily. Of the 100 members, five steroid receptors are known targets of EDCs; they are AR, estrogen receptor (ER), glucocorticoid receptor (GR), mineralocorticoid receptor (MR), and progesterone receptor (PR) [18].

The estrogen receptor is one of the important endocrine targets involved in cancer and other diseases [19,20]. ER has two major isoforms: ERα and ERβ. They are encoded in the genes that locate on different chromosomes and are the targets of EDCs. Lot of experimental data on estrogenic activity have been generated [21,22,23,24] and many in silico prediction models have been developed [25,26,27,28,29,30,31,32,33]. Estrogen receptors play a major role in the metabolic regulations [18]. The isoforms modulate the insulin sensitive tissues, indicating that estradiol (E2) involved in the regulation of insulin sensitivity gene and glucose uptake [34,35,36,37]. The estrogen receptor α plays an important role in the maintenance of adipose tissues [38]. The dysfunction of adipose tissue leads to fat accumulation as well as an increased risk of diabetes mellitus, liver disease, hypertension, and cancers [39]. Certain drugs can selectively modulate the function of ERs and interact with the target genes, represent a promising frontier in diabetes mellitus research [40].

In addition, EDCs have been reported to promote adipogenesis through modulation of peroxisome proliferator receptors (PPARs), which act as a transcription factors and control the expression of genes involved in lipid, glucose and cholesterol metabolism [41]. Three members of PPARs, PPARα, PPARβ and PPARγ have different tissue distribution and biological functions. Peroxisome proliferator receptors α and β play a major role in regulation of energy homeostasis through activation of fatty acid oxidation pathway [42]. Peroxisome proliferator receptor γ is expressed predominately in adipose tissues and promote adipogenesis through improving insulin sensitivity [43]. Some EDCs such as phthalate interfere with PPARs that regulating metabolism, associating with metabolic diseases such as obesity and diabetes mellitus [44,45,46].

The hypothalamic pituitary adrenal axis controls the production of glucocorticoid hormones through the adrenal cortex. Glucocorticoid binds in the active site of GRs and MRs in different target tissues [47]. Excess level of cortisol disturbed the metabolic intermediate by altering the insulin resistance, cholesterol level, etc., which leads to diabetes mellitus. In 1992, Weaver et al. reported that the mutation in GR gene leads to metabolic syndrome [48]. The glucocorticoid receptor is one of the extensively studied nuclear receptors due to its roles in various physiological processes such as glucose balance, fat distribution, and normal growth and development [49]. The glucocorticoid receptor regulates the action of glucocorticoid. Glucocorticoid binds GR and regulates the glucose metabolism in various tissues such as liver, pancreas, and adipose tissues.

Aldosterone is the primary endogenous member of the mineralocorticoid hormones. Aldosterone acts on the MR to enhance the reabsorption and excretion of sodium and potassium in the kidney. The function of MR is to maintain blood pressure as well as electrolyte homeostasis through the distal nephron [50]. Aldosterone and cortisol can bind MR to stimulate its functions. The mineralocorticoid receptor agonists decrease the effect of renin-angiotensin system (RAS) which elevates blood pressure, improves insulin resistance, and prevents nephropathy. Eplerenone has shown an improved specificity towards MR and acts as a partial antagonist [51]. Recently, eplerenone was approved as a drug for hypertension [52].

High level of progesterone, an endogenous steroid hormone, leads to development of glucose abnormalities in the second trimester of pregnancy [53]. Hence, progesterone might play a crucial role in the development of gestational diabetes mellitus and also specific effects in diabetic renal complications [54]. Progesterone is important to activate PR which has two forms, PRA and PRB, with different molecular weights [55]. The progesterone receptor is mainly present in the distal tube cells of kidney. Diabetes mellitus in women might be treated with either progesterone or a combination of progesterone and estrogen [56,57].

The androgen receptor plays an important role in male sexual development and regulates gene expression in various tissues. Lack of testosterone in men could lead to development of T2DM and hypogonadism [12,58]. The androgen receptor can be activated by any synthetic or natural androgens. Excess androgens in women induced T2DM and insulin resistance [12]. Testosterone is mainly mediated by AR; the ligand activated transcriptional factor. Tissue selectivity of AR alters fat metabolism and glucose level in females and males. Binding of testosterone in the active site of AR plays a role in energy homeostasis/glucose control and leads to metabolic dysfunction because of its tissue selectivity [12]. Most of these ligand-dependent transcriptional factors regulate various pathways and play a major role in cancer to metabolic diseases [59].

## 3. Androgenic Activity

**Figure 1:**
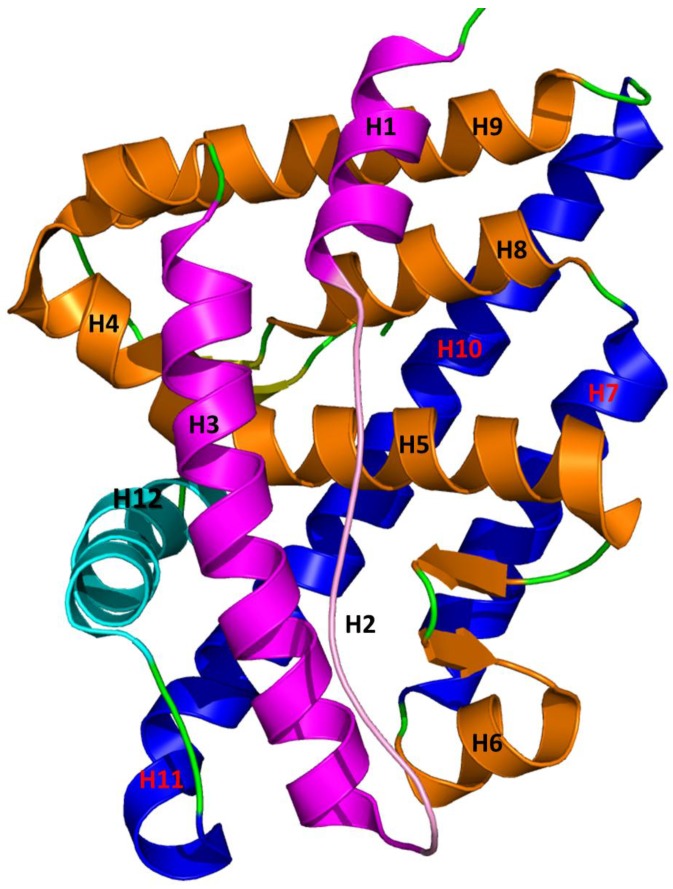
Three-dimensional structure of ligand binding domain of wild-type androgen receptor (WT-AR). The helices in ligand binding domain were arranged in a three-layered antiparallel α-helical sandwich fold. The first layer consists of H1 and H2 which are colored in magenta; the second layer contains H4, H5, H8, H9, and the first β-sheet which are colored in orange; and the third layer has H10 and H12, which are colored in blue and cyan, respectively. H12 acts as a lid in the entrance of the ligands binding pocket.

The androgen receptor is a soluble nuclear receptor protein. The human AR gene is located on X-chromosome and contains 2757 nucleotides that encode the AR protein [17,60,61]. The AR gene contains eight exons with various sizes [62]. Similar to other nuclear receptors, AR consists of three main functional domains: N-terminal domain (NTD), DNA binding domain (DBD), and ligand binding domain (LBD) or C-terminal domain (CTD). The highly conserved DBD is connected to LBD through a hinge region. The LBD shows a similar structure with those of other nuclear receptors [63]. The tertiary structure of AR contains 11 α-helices and two short β-turns that form an antiparallel α-helical sandwich with three layers (Figure 1) [61,64]. The H2 is not present in AR LBD which makes AR different from other steroid receptors. H1 and H3 form the first layer of LBD; H4 and H5 form the first β-turn; H8 and H9 form the middle layer; and H6, H7, H10 and H11 form the third layer of α-helical sandwich. The hydrophobic region of the ligand binding pocket (LBP) consists of H5, H3, and H10 and H11. H12 acts as a lid in front of the LBP due to the binding of agonist which stabilizes LBD and makes activation function 2 (AF2) site suitable for binding of substrate protein to activate AR [65,66]. The surface of AF2 is mainly a hydrophobic region formed by the H3, loop 3–4, H4, and H12 [67]. AF2 site is highly conserved and crucial for co-activator recruitment.

**Figure 2:**
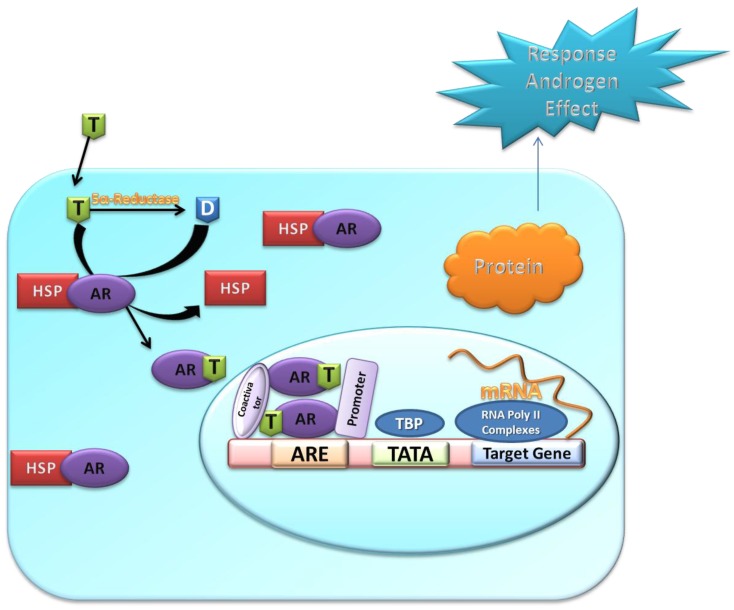
Illustration of AR signaling path. T, Testosterone; D, dihydrotestosterone; HSP, heat shock proteins; AR, androgen receptor; ARE, androgen response elements. When T enters the cell, it either is converted into the more potent D by 5α-reductase and binds to the ligand binding pocket of AR. AR–agonist complex is then translocated into the nucleus with help of other proteins to form AR-dimer that binds to ARE of DNA and co-regulator proteins to initiate transcription of AR.

The mechanism of androgenic activity consists of multiple steps that are illustrated in Figure 2. The androgen receptor resides in cytoplasm as an inactive form that forms a complex with various chaperones. When androgen enters the cell it either directly binds the inactive AR or is converted into metabolite, dihydrotestosterone (DHT), that binds the inactive AR. Due to binding of androgens such as testosterone, DHT, or other AR agonists, AR undergoes a conformational change which leads to the dissociation of chaperones and other proteins from the inactive AR [68]. The dissociated AR monomer (active AR) is then translocated to the nucleus and forms AR homodimer that acts as a transcriptional regulator to regulate the gene expression by binding to the specific androgen response element (ARE) site in DNA [69]. The AR dimer forms signaling complexes with co-regulated proteins to enhance or depress transcription of the target gene.

## 4. Metabolism of Androgens

Androgen is a steroid derived sex hormone. It plays a major physiological role in male. Testosterone and DHT are the well-known and important androgens. Testosterone is the major circulating androgen and is synthesized from cholesterol in several steps. Initially, steroidogenic acute regulatory (StAR) protein transfers cholesterol from outer mitochondrial membrane into inner membrane. Subsequently, cholesterol side-chain cleavage (P450scc) enzyme cleaves the side chain of cholesterol [70]. This conversion is the rate limiting step in the biosynthesis of testosterone.

The 5α-reductase catalyzes the conversion of testosterone into DHT. Dehydrotestosterone is a metabolic product that is more active than testosterone [71]. The androgen receptor is activated by androgen to mediate most of the AR biological effects. Testosterone is the primary androgen required for the bioactivity of AR. However, few androgenic tissue specific activities required DHT.

## 5. Interaction between Agonist/Antagonist and the Androgen Receptor

**Figure 3:**
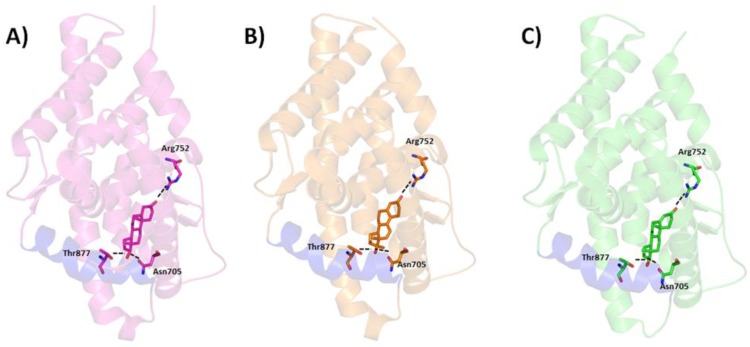
Binding of AR agonists dihydrotestosterone DHT (**A**), Testosterone (**B**), and R1881 (**C**) in the active site of AR ligand binding pocket. The agonists were drawn in stick model and the protein in ribbon model. The important residues were displayed in stick model. The black dotted lines represent the hydrogen bond interactions between the agonists and critical residues in the active site of AR ligand binding domain.

There are increasing evidences that androgen plays a major role in prostate cancer. The androgen receptor is mainly activated by androgens (agonist) such as testosterone, DHT, synthetic androgen etc. Agonist binding in the LBP (active site) of AR-LBD holds H12 to maintain the active conformation of AR [72]. However, binding of an antagonist moves H12 from the original position and distorts the formation of AF2 site. AF2 site is important for binding of target proteins. Complexes of wild type AR-LBD with agonist and mutated AR-LBD with agonist and antagonist were crystalized and their structures were determined. No crystal structure of wild type androgen receptor ligand binding domain (WT-AR-LBD) with antagonist has been reported. The 3D structures of AR-agonist complexes revealed that the agonists mainly interact with the hydrophobic amino acids in the active site of AR. The steroid scaffolds of agonists mainly interact with AR through van der Waals interactions. The remaining polar amino acids form hydrogen bonds with the agonists. The binding pattern of the three agonists (DHT [73], metribolone (R1881) [74], and testosterone [75]) in AR was elucidated by analysis of the crystal structures, indicating similar hydrogen bond interactions with amino acids N705, R752, and T877 (Figure 3).

Antiandrogens or AR antagonists are a competitive binder for androgens in AR. They are synthetic compounds which bind in the androgen binding site of AR. An antiandrogen inhibits AR activity by blocking binding of androgen in the active site of AR. Binding of an antagonist in the active site of mutated AR showed a similar structural changes and binding interaction as an agonist binding in wild type AR. Due to the absence of wild type AR-LBD with antagonist crystal structure, there is no strong evidence to propose the binding orientation of antagonists in AR [17]. Binding of an antagonist in the LBP of AR had different conformational changes in the AF2 site of AR [76]. The conformational changes occurred in the AF2 site of AR is not suitable for co-activator proteins to bind AR to initiate transcriptional signaling. Binding of antiandrogen represses AR gene expression and leads to the cancer [77]. Drug resistance is another challenging to design the AR antagonists [78]. Initially, most of the AR antagonists suppress the tumor growth, but fail to suppress in long-term treatment [79,80,81].

## 6. Androgenic Compounds Associated with Diabetes Mellitus

Binding of androgens to AR converts inactive AR to its active form. The active AR regulates various target genes by interacting with different coregulatory proteins. There are growing evidences to support the role of androgens in diabetes mellitus [82]. The deficiency of testosterone promotes metabolic syndrome, T2DM, vascular disease, etc. [83,84,85,86,87,88,89]. Increasing level of testosterone decreases insulin resistance which significantly reduces the risk of diabetes mellitus [90,91]. Binding of testosterone with AR activates AR signals, which play an important role in testosterone potentiation of glucose-stimulated insulin secretion (GSIS). Deficiency of GSIS due to the lack of androgen leads to T2DM [82]. The glucose concentration was increased due to the supplementation of testosterone. The AR in liver cells binds testosterone to increase the insulin sensitivity in hepatocytes. Androgen might play different roles in various disorders caused by diabetes mellitus.

**Figure 4:**
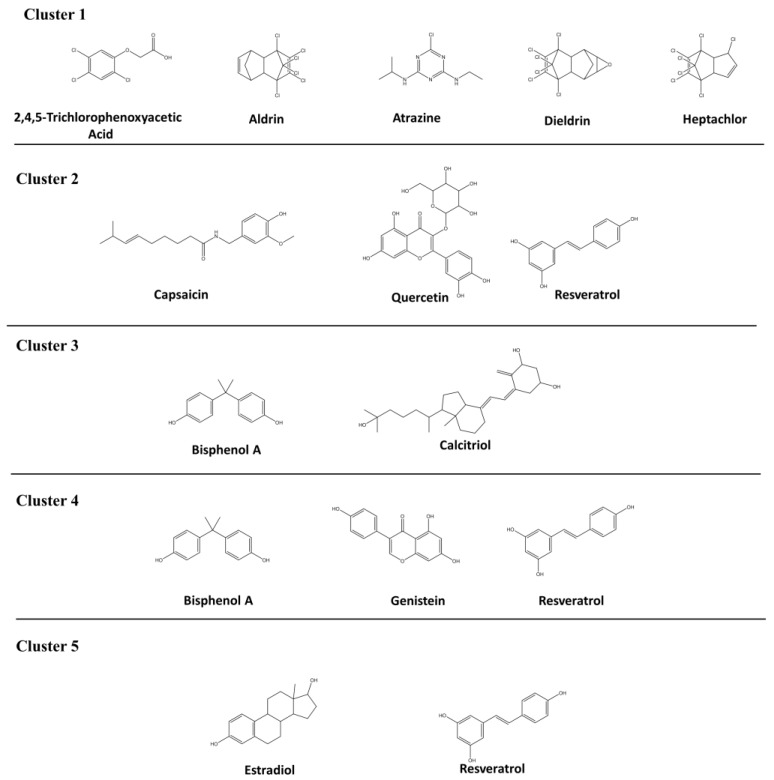
Two-dimensional structures of androgenic activity compounds associated with diabetes mellitus.

AR associated diseases and chemicals that interact with AR were downloaded from Comparative Toxicogenomics Database (CTD,). Comparative analysis on the diseases associated with AR and the chemicals interacted with AR found that 13 androgenic chemicals are associated with diabetes mellitus. The 13 androgenic compounds were clustered into five groups based on the medical subject headings (MESH) ID (Table 1). The 2D structures of the 12 androgenic compounds related to diabetes mellitus are depicted in Figure 4. Clusters 1 and 2 contain five and three compounds related to diabetes mellitus and diabetes mellitus experimental, respectively. T1DM and T2DM contain two and three compounds present in Clusters 3 and 4, respectively. Cluster 5 represents diabetic nephropathies having two compounds. Two compounds (resveratrol and bisphenol A) are associated with more than one MESH codes related to diabetes mellitus.

### 6.1. Androgenic Compounds and Diabetes Mellitus

Cluster 1 has organochlorine insecticides and pesticides that are potential EDCs in the environment. These chemicals disrupt the function of AR by mimicking its natural hormones such as testosterone. Few pesticides and their metabolites bind in the ligand binding pocket of AR, altering AR normal functions. These chemicals act as antagonists in vitro. Fang et al. reported the relative binding affinity of 2,4,5-trichlorophenoxyacetic acid(2,4,5-TP), aldrin, and heptachlor were 0.0007, 0.0096, and 0.0133, respectively, in the competitive binding assay [106]. 2,4,5-Trichlorophenoxyacetic acid, aldrin and heptachlor are weak or moderate binders for AR. The chloro group in the pesticides had shown more significant role in binding AR than ER. Atrazine is a chlorotriazine herbicide and reported as a non-binder for AR in competitive binding assay. However, atrazine had shown a positive result in the endocrine disrupter screening. Hence the Endocrine Disruptors Testing Advisory Committee (EDSTAC) considered it as an endocrine disruptor [107]. Dieldrin, chlorinated hydrocarbon insecticide, acted as an antiandrogen in in vitro studies by reducing the intake and retention of androgen such as 5-DHT to its receptor.

Increasing use of pesticides in the environment has been found associated with the risk of diabetes mellitus. Long term exposure of pesticides can increase the risk of diabetes mellitus [92,93]. Organochlorines and dioxins are two pesticides that have been found to be associated with the risk of diabetes mellitus [108,109,110,111]. Aldrin, dieldrin, and heptachlor are organochlorine cyclodienes which share a similar structure associated with the increased risk of diabetes mellitus [94]. 2,4,5-Trichlorophenoxyacetic acid is a contaminated product of dioxins associated with diabetes mellitus that shows a different effect in women and men. It was proven that the glucose metabolism was disturbed due to the exposure of pesticides [112,113] which results in elevation in insulin and glucose concentration in blood [114,115,116]. Exposure of the pesticides and their metabolites in the environment plays a major role in the human health and disease conditions. The pesticides disrupt the natural hormone functions, which leads to the adverse health effect in human [94]. Long term exposure of these pesticides increased the diabetes mellitus in human. The organochloro pesticides are widely used in the environment and, thus, could increase the chance of diabetes mellitus in human.

### 6.2. Androgenic Compounds and Diabetes Mellitus Experimental

“Diabetes mellitus experimental” is a descriptor in the National Library of Medicine’s controlled vocabulary thesaurus, MeSH (Medical Subject Headings). It is designated as “Diabetes mellitus induced experimentally by administration of various diabetogenic agents or by pancreatectomy”. Capsaicin and clioquinol play a role in diabetes mellitus through various pathways.

Capsaicin is a non-pungent capsaicin analog which modifies or alters the secretion of insulin [95]. It increases proliferation and decreases apoptosis of beta cells through insulin-like growth factor 1 (IGF-1) signaling [117]. Quercetin, a natural polyphenolic flavonoid, mostly presents in onions, green vegetables, citrus fruits, etc. Quercetin is an antidiabetic compound targeting the oxidative stress and hyperglycemia. Hyperglycemia is a basic feature of T2DM which is mainly related to oxidative stress [96]. Quercetin protects B cells and depresses aldose reductase. Aldose reductase is an enzyme which converts glucose to sorbitol. Elevated concentration of sorbitol leads to various types of diabetes mellitus. It was proven that resveratrol has antidiabetic property, decreasing blood glucose level and elevating level of hemoglobin and plasma insulin in diabetic rats [97].

### 6.3. Androgenic Compounds and Type 1 Diabetes Mellitus

Bisphenol A (BPA), a diphenylmethane derivative, is an antagonist for AR. Bisphenol A is one of the endocrine disruptors which bind in the ligand binding pocket of AR and alter its functions. Bisphenol A had shown AR-antagonistic activity in reporter gene assay with an IC_50_ value of 5900 nM. Some BPA derivatives also showed antagonistic effect towards AR [118]. Calcitriol is an active form of vitamin D3. It regulates calcium homeostasis, differentiation and proliferation of various types of cells, and bone metabolism. Calcitriol plays an important role in prostate cancer by reducing androgen glucuronidation [119].

Type 1 diabetes mellitus (previously called insulin-dependent diabetes mellitus or juvenile or childhood-onset diabetes mellitus) is characterized by deficient insulin production and requires daily administration of insulin [120]. It accounts for 5–10% of diabetes mellitus and is caused by cellular-mediated autoimmune impairment of the pancreatic β-cells. Type 1 diabetes mellitus is defined by the following autoimmune markers: islet cell autoantibodies, autoantibodies to insulin, autoantibodies to GAD (GAD65), autoantibodies to the tyrosine phosphatases islet antigen 2 (IA-2) and IA-2β, and autoantibodies to zinc transporter 8 (ZnT8) [121]. The cause of T1DM is not known and it is not preventable with current knowledge. Numerous clinical studies are being conducted to test various methods of preventing T1DM in those with evidence of autoimmunity.

The chemicals in Cluster 3 are androgenic and play an important role in T1DM [98,99]. Bisphenol A is an endocrine disruptor highly exposed or used in our daily activities. Bisphenol A used as a polycarbonate plastic in most of the food and beverage containers. Recent study proved the association of BPA exposure with T1DM by accelerating insulitis. Exposure of BPA can raise the risk of T1DM through increasing severity of pancreas endocrine tissues, insulitis. Bodin et al. reported that long-term exposure of BPA accelerated development of T1DM in mice [98]. Bisphenol A increases apoptosis in T1DM in many pathways such as suppressing B-cell lymphoma 2 (Bcl-2), an anti-apoptotic protein, and inducing reactive oxygen species (ROS) in hepatocytes. Calcitriol, also known as 1,25-dihydroxy-vitamin D3, is the active metabolite of vitamin D and plays a role in anti-inflammatory diabetes mellitus. It is involved in the immune system regulation and protects destruction of pancreatic islet cells induced by cytokines [99]. Calcitriol slows down the progress of diabetes mellitus through inflammation. Intake of calcitriol did not alleviate beta cell function or hyperglycemia and plays a role in the prevention of T1DM [98].

### 6.4. Androgenic Compounds and Type 2 Diabetes Mellitus

Bisphenol A is a polycarbonate plastics present in many consumer products like water bottles and food. Bisphenol A is androgenic and was found associated with both T1DM [98] and T2DM [100]. In addition to diabetes mellitus experimental as mentioned above, resveratrol was found associated with T2DM. Genistein is an isoflavone and an inhibitor for angiogenesis. It inhibits cell growth in many cancers. Geneistein modulates expression of AR and its transcriptional activity in prostate cancer. Genistein is a weak binder of AR and ER [24]. The relative binding affinity of genistein to AR was 0.0036. Even in presence of DHT, genistein inhibits AR activity. Genistein showed 1400-fold less active than flutamide and 10,000-fold less active than DHT based on in vitro reporter gene assay [122].

Type 2 diabetes mellitus (formerly called non-insulin-dependent or adult-onset diabetes mellitus) results from the body’s ineffective use of insulin. Type 2 diabetes mellitus, a common disease, accounts for 90–95% of the people with diabetes mellitus around the world and is estimated to affect 380 million people worldwide by 2025 [120]. Type 2 diabetes mellitus encompasses individuals who have insulin resistance and usually relative (rather than absolute) insulin deficiency [120]. There are various causes of T2DM and most patients with D2DM are obese [120]. The risk of T2DM is determined by an interplay of genetic and metabolic factors. The risk of developing T2DM increases with age, obesity, and lack of physical activity. It has been reported that T2DM occurs more frequently in women with prior gestational diabetes mellitus (GDM), in those with hypertension or dyslipidemia, and in certain racial/ethnic subgroups (African American, American Indian, Hispanic/Latino, and Asian American) [120].

Exposure to BPA leads to insulin resistance which results in T2DM through the highly expressed pancreatic β cells [100]. Bisphenol A plays a role in both T1DM and T2DM. Bisphenol A induces apoptosis and destroys β-cells in pancreases T1DM. In addition, it causes insulin resistance by over expression of pancreatic β-cells to develop T2DM. Bisphenol A can alter the biosynthesis of insulin and the β-cell secretion in pancreatic cells. Resveratrol can activate and inhibit functions of major target proteins related to cancer and diabetes mellitus. In T2DM, the antidiabetic effect of resveratrol is mediated by the class III histone deacetylases (HDAC) protein, sirtuin 1 (SIRT1) [103]. Resveratrol activates NAD(P)H oxidase by inhibiting tumor necrosis factor α (TNF-α) to restore the endothelial function in T2DM [102]. Resveratrol had shown a potent antidiabetic activity in T2DM in rodent models. Geneistin is one of the major bioactive isoflavones present in soy. Geneistin is converted into genistein by glycosidase. Genistein enhanced the lipid metabolism and modulated hepatic glucose to exert its antidiabetic effects in mice [101].

### 6.5. Androgenic Activity Compounds and Diabetic Nephropathies

In addition to diabetes mellitus experimental and T2DM, resveratrol was also found associated with diabetic nephropathies (DN). Estradiol, a steroid estrogen, had a relative binding affinity of 0.758 in the AR competitive assay [106]. Estradiol increases activity of mutant AR by expanding its ligand binding site. Activation of the mutant AR leads to prostate cancer.

Diabetic nephropathy is a well-described complication of diabetes mellitus and is one of the microvascular complications of the kidney commonly due to T1DM, and occasionally from T2DM. Diabetic nephropathy is characterized by glomerular hypertrophy, proteinuria, decreased glomerular filtration, and renal fibrosis resulting in the loss of renal function [123]. Diabetic nephropathy is considered as a multifunctional degenerative disorder where the mechanism of disease progression involves various signaling cascades. Several mechanisms have been proposed, including hyperglycemia-induced renal hyper filtration and renal injury, advanced glycation end-products (AGEs)-induced increased oxidative stress, activated protein kinase C (PKC)-induced increase in production of cytokines, chemokines, and different inflammatory and apoptotic signals [124].

Estradiol decreases activity of reticular activating system (RAS) system by reducing renin levels and increasing angiotensin II type 2 receptors (AT2Rs) which are associated with DN [125]. Lowering the circulating level of estradiol as well as the imbalance between renal and estrogen receptors leads to DN. Increasing the level of estradiol might lower diabetic effects in renal diseases. Estradiol regulates many pathways associated to DN: (i) controls renal RAS by lowering its activity through downregulating AT1Rs and lowering the renin level; (ii) reduces oxidative stress by lowering oxidase activity of NADPH; (iii) attenuates inflammation by reducing the acute and chronic inflammation in their target tissues; and (iv) reduces hyperglycemia and increase protein degradation of extracellular matrix (ECM). Resveratrol had shown beneficial effect in human against various diseases like cardiovascular, cancer, and diabetes mellitus [105]. Resveratrol plays a major role in various types of diabetes mellitus like DM experimental, T2DM, and DN.

## 7. Conclusions

Diabetes mellitus is recognized as one of the risk factors in cancer development. Endocrine disrupting chemicals mediated through AR have been found associated with diabetes mellitus. However, understanding of the mechanisms of androgenic EDCs causing diabetes mellitus is limited. Studies to ascertain the causality of diabetes mellitus by androgenic EDCs are expected in the future. As only few of the known androgenic EDCs have been assessed for their risks to diabetes mellitus in clinical and animal studies, the risk of diabetes mellitus for the remaining known androgenic EDCs should be evaluated to better protect the public health.

## Figures and Tables

**Table 1:** Androgenic compounds and the associated types of diabetes mellitus.

Cluster	MESH ID	Diabetes Mellitus Type	Description	Chemical [reference*]
1	D003920	Diabetes mellitus	Production of excess glucose level in the blood for a long term	2,4,5-Trichlorophenoxyacetic acid [92,93], aldrin [94], atrazine [93,94], dieldrin [92,94], heptachlor [94]
2	D003921	Diabetes mellitus, experimental	Experimentally induces diabetes mellitus by various diabetogenic agents	Capsaicin [95], quercetin [96], resveratrol [97]
3	D003922	Diabetes mellitus, type 1	Insulin-dependent diabetes mellitus—failed to produce enough insulin	Bisphenol A [98], calcitriol [99]
4	D003924	Diabetes mellitus, type 2	Non-insulin-dependent diabetes mellitus—Resistance to insulin production	Bisphenol A [100], genistein [101], resveratrol [102,103]
5	D003928	Diabetic nephropathies	Diabetes mellitus leads to kidney failure	Estradiol [104], resveratrol [105]

* Reference collected from Comparative Toxicogenomics Database (CTD).
